# Leiomyomatosis peritonealis disseminata associated with appendiceal endometriosis: a case report

**DOI:** 10.1186/s13256-015-0637-1

**Published:** 2015-07-28

**Authors:** Woo Yong Lee, Ji Hyun Noh

**Affiliations:** 1 Department of Surgery, Seoul Paik Hospital, Inje University College of Medicine, 9, Mareunnae-ro, Jung-gu, Seoul 100-032 Korea; 2 Department of Obstetrics and Gynecology, Ilsan Paik Hospital, Inje University College of Medicine, 170, Juhwaro, Ilsanseogu, Gyeonggido 411-706 Korea

**Keywords:** Appendix, Endometriosis, Leiomyomatosis peritonealis disseminata (LPD)

## Abstract

**Introduction:**

Leiomyomatosis peritonealis disseminata is a very rare benign condition of the peritoneal cavity that may mimic peritoneal carcinomatosis or metastatic leiomyosarcomas. It mainly develops in association with pregnancy, but is also rarely associated with endometriosis.

**Case presentation:**

A 31-year-old Asian woman presented to our hospital with abdominal pain in the right lower quadrant. Her abdominopelvic computed tomography scan showed a 1.2cm-sized nodule at the appendiceal tip, but no other abnormal findings. We suspected acute appendicitis and performed an exploratory laparoscopy. Her appendix was enlarged at the tip portion. Also noted were blood-colored fluid collections in her pelvic cavity and bilateral ovarian cysts. Additionally, several small whitish firm solid nodules, ranging from 0.5 to 1.0cm in size, were present on her pelvic peritoneum. Her histological examination confirmed that the endometriosis of her appendix coexisted with leiomyomatosis peritonealis disseminata.

**Conclusions:**

We report a case involving a 31-year-old woman with acute symptoms of endometriosis of the appendix associated with leiomyomatosis peritonealis disseminata. Appendiceal endometriosis with leiomyomatosis peritonealis disseminata presenting as acute appendicitis is extremely rare. To the best of our knowledge, this is the first such case reported in the literature.

## Introduction

Acute appendicitis is the most common surgical emergency encountered by general surgeons. Endometriosis is a common gynecologic disorder characterized by the presence of endometrial glands and stoma outside the uterine cavity and musculature [1]. Leiomyomatosis peritonealis disseminata (LPD) is a rare disease characterized by multiple subperitoneal foci or peritoneal proliferation of benign tumorous tissue mainly comprising benign smooth muscle cells. It is most often associated with pregnancy and prolonged exposure to oral contraceptive pills. The combination of appendiceal endometriosis and LPD presenting as acute appendicitis is extremely rare. To the best of our knowledge, this is the first such case reported in the literature.

## Case presentation

A 31-year-old Asian woman presented to our hospital with abdominal pain in her right lower quadrant. She had no fever, nausea, vomiting, anorexia or unusual vaginal discharge. Her menstruation cycle was regular, her volume of menstrual fluid was normal and she had no dysmenorrhea. She had no history of pregnancy. Her last menstrual period was four days prior to presentation. She had a history of a benign ovarian cyst, uterine leiomyoma and endometriosis. She had undergone a laparoscopic ovarian cystectomy six years previously and a laparoscopic uterine myomectomy one year previously. She had been intermittently taking oral contraceptive pills since her most recent post-operative period.

**Fig. 1:**
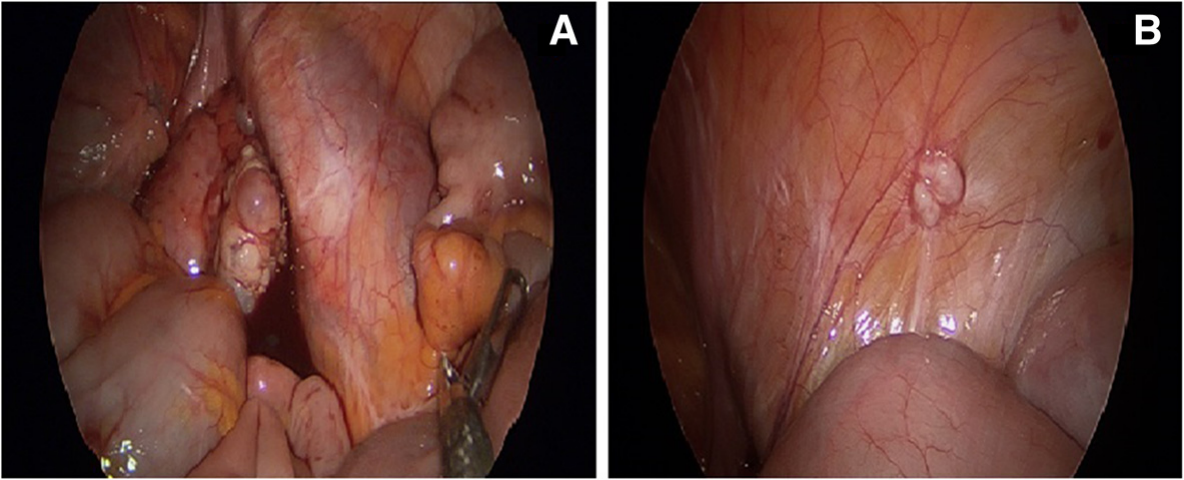
Laparoscopic views showed an enlarged appendiceal tip, a bloody fluid collection in the pelvic cavity, multiple bilateral ovarian cysts (**a**) and several small whitish firm solid nodules (**b**), ranging from 0.5 to 1.0cm in size, on her pelvic peritoneum

She exhibited tenderness at McBurney’s point, but her abdomen was soft and flat. Her vaginal examination and pelvic sonography revealed no abnormal findings. Her white blood cell count was 4280/mm^3^ with 57.0% segmented neutrophils, and her C-reactive protein level was 0.1mg/dL. Her urine analysis and abdominal X-rays showed no significant findings. Her abdominopelvic computed tomography scan demonstrated a 1.2cm-sized nodule at the appendiceal tip, but no other abnormal findings. We suspected acute appendicitis and performed an exploratory laparoscopy. Her appendix was enlarged at the tip portion. Also noted were blood-colored fluid collections in her pelvic cavity and bilateral ovarian cysts (Fig. 1a). Also observed on the pelvic peritoneum were several small whitish firm solid nodules, ranging from 0.5 to 1.0cm in size (Fig. 1b). We performed a laparoscopic appendectomy and obtained specimens of the peritoneal nodules for biopsy.

**Fig. 2:**
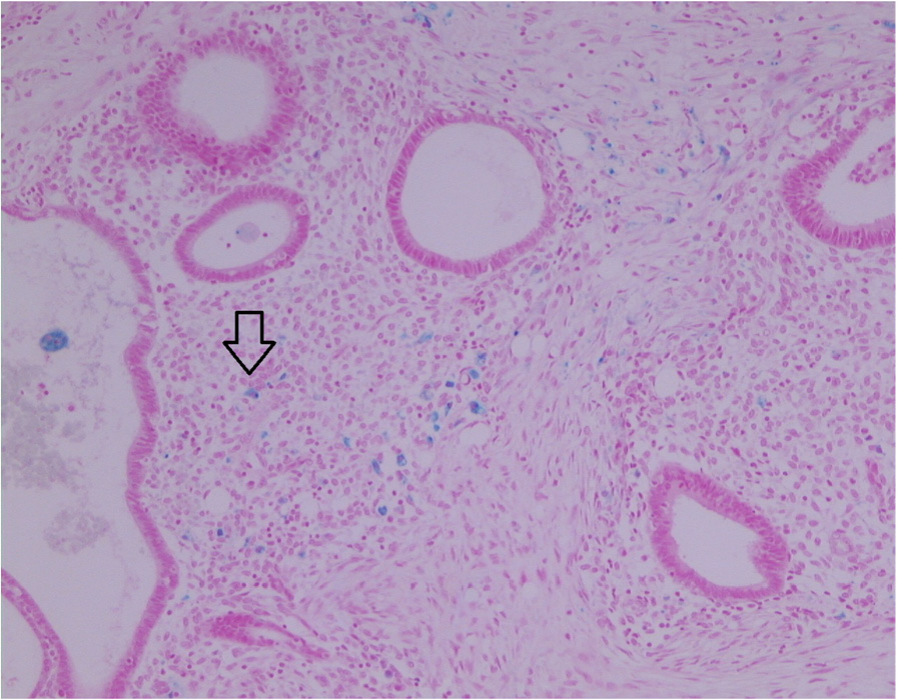
Histological examination of biopsy. A histological examination revealed foci of ectopic endometrial glands and stroma with some hemosiderin-laden histiocytes (arrow) in the subserosa and the muscularis propria of the appendiceal tip (Prussian blue, ×100)

**Fig. 3:**
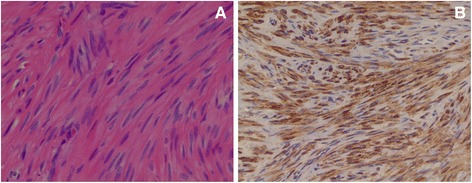
Photomicrograph of the peritoneal nodules, exhibiting interlacing bundles to fascicular proliferation of bland-looking spindle smooth muscle cells. **a** Hematoxylin & eosin, ×400. **b** Desmin, ×200

Macroscopically, her appendix was 4.0×0.5cm in size and exhibited an enlarged tip. The appendiceal serosa was mildly inflamed. A histological examination revealed foci of ectopic endometrial glands and stroma, embedded in fibrous stroma, with patchy aggregates of exuberant hemosiderin-laden histiocytes in the subserosal area and the muscularis propria of the appendiceal tip portion, confirming a diagnosis of appendiceal endometriosis (Fig. 2). No suppuration, abscess formation or neoplasms were seen in the appendectomy specimen. The peritoneal excisional biopsy specimens were characterized by two circumscribed, but nonencapsulated, solid nodules, comprising of interlacing bundles of a fascicular proliferation of bland-looking, spindle-shaped, mature, smooth muscle cells (Fig. 3a), which were diffusely strongly immunoreactive for desmin (Fig. 3b). These peritoneal tumors exhibited no histological features of malignancy, with absence of any tumor cell coagulation necrosis, mitotic activity or nuclear atypia. As a result, they were diagnosed as LPD.

She had an uneventful clinical course and was discharged from our hospital on post-operative day five. She was followed up on for one year post-operatively, and no complications or abnormalities were observed. Her follow-up period is currently ongoing.

## Discussion

Endometriosis is a common benign gynecologic disorder of the endometrial glands and stroma located outside of the endometrium [1]. Endometriosis usually develops in the genital organs and pelvic peritoneum. It may also occur in the gastrointestinal system, greater omentum, mesentery, liver and in operation scars, but rarely in other organs [2]. Endometriosis can develop anywhere in the gastrointestinal system, from the small intestine to the anus; the rectosigmoid region is the most common gastrointestinal site (72% of cases), followed by the recto-vaginal septum (13%), small bowel (7%), cecum (4%) and appendix (3%) [2–4].

Appendiceal endometriosis was first described in 1860 by Von Rokitansky [5]. According to several studies, the incidence of appendiceal endometriosis is between 0.8 and 22.0% [6–8]. Appendiceal endometriosis is classified into primary and secondary forms. The primary form is characterized by the pathological presence of endometriosis within the appendix without extra-appendiceal endometriosis, and the secondary form is defined by the pathological presence of internal and/or external endometriosis. Most studies have described an analogy between appendiceal endometriosis and tubo-ovarian endometriosis. Most patients with appendiceal endometriosis have menstrual irregularities and uterine leiomyomas, and similar cases of primary appendiceal endometriosis have been reported [2, 9, 10]. Appendiceal endometriosis can be categorized into four groups, according to the associated symptoms: acute appendicitis, invaginated appendix, atypical appendiceal endometriosis (symptoms such as abdominal colic, nausea and melena) and asymptomatic appendiceal endometriosis. The most common type is acute appendicitis, and symptoms usually occur during menstruation [2, 11, 12]. Acute appendiceal inflammation can be caused by partial or complete luminal occlusion by the endometrioma [13]. Another proposed mechanism involves endometrial hemorrhage within the seromuscular layer of the appendix, followed by edema, obstruction and inflammation. Pain in the right lower abdominal quadrant is one of the most common symptoms, and one third of affected patients present with a typical appendiceal symptom [2].

Our patient underwent an appendectomy for the treatment of appendicitis with right lower quadrant pain. Several nodules were found in her peritoneal cavity, and a biopsy was performed. The histological features of the peritoneal nodules were consistent with LPD, and those of the appendix were consistent with appendiceal endometriosis. These results appeared to be associated with her history of ovarian cystectomy by endometriosis and myomectomy.

LPD is a rare disease characterized by multiple peritoneal nodules on the omentum or any surface of the peritoneal cavity. Although LPD was first described by Wilson and Peale in 1952, it was first clearly delineated and named by Taubert *et al*. in 1965 [14, 15]. About 50 cases have been reported to date, and most have occurred in patients of reproductive age. Although the etiology of LPD is unclear, it is thought to originate from metaplasia of submesothelial, multipotential mesenchymal cells. The developing leiomyomatous nodules probably arise from Mueller’s epithelium, which is distributed throughout the subperitoneal mesenchyme. The pathogenesis is unknown, but the Mullerian derivatives proliferate along lines of myofibrous differentiation, according to individual predisposition and excessive hormonal stimulation [16]. More than half of affected patients were reportedly pregnant or taking oral contraceptive pills at the time of diagnosis [17]. Associations of LPD with granular cell tumor of the ovary, endometrial adenocarcinoma, clear cell carcinoma of the ovary and estrogen-secreting ovarian fibrothecoma have also been found [14, 18, 19]. Estrogen and progesterone receptor expression was found in nearly all cases [20]. In some cases, LPD was found in post-menopausal women who had undergone total hysterectomy several years before and had received no subsequent hormonal therapy [16, 21, 22]. LPD is associated with myomectomy, and reportedly occurred in two patients after laparoscopic- assisted myomectomy and hysterectomy [23]. The findings in our case report further confirm an association between LDP and prolonged exposure to estrogen.

An association between LPD and endometriosis was first reported in 1980 [24]. Toriyama *et al*. reported LDP coexisting with endometriosis within the same lesions [25]. This association with endometriosis suggests that LPD may originate from the metaplasia of submesothelial, multipotential mesenchymal cells. However, this association has been reported in only a few cases throughout the literature [26, 27]. As in our case report, previously reported cases of LPD seemed to be associated with multiple factors.

LPD is generally benign and has a good prognosis. Malignant change of LPD is very rare; the time from first diagnosis to malignancy varies from several months to several years. Many patients with malignant change of LPD exhibit no exogenous estrogen stimulation, estrogen and progesterone receptor negativity within the tumor, and no leiomyoma. Our patient was classified as belonging to the high-risk group according to Bekker *et al*. [28], and required close observation during the first year. No treatment guidelines for LPD have been established. In most cases, spontaneous tumor regression occurs after hysterectomy and discontinuance of exogenous estrogen stimulation, such as pregnancy or oral contraceptives.

## Conclusions

We suggest that the optimal treatment for LPD be determined by the patient’s age, associated diseases, severity of LPD and chief symptoms. The contributing factors for LPD with appendiceal endometriosis in our patient appeared to be previous myomectomy, ovarian cystectomy by endometriosis and a history of taking oral contraceptive pills. In conclusion, we have described a rare case of LPD coexisting with appendiceal endometriosis, which presented as acute appendicitis.

## Consent

Written informed consent was obtained from the patient for publication of this case report and accompanying images. A copy of the written consent is available for review by Editor-in-Chief of this journal.

## Abbreviation

LDP: Leiomyomatosis peritonealis disseminata
